# Supplementary material for: DiNeR: a Differential graphical model for analysis of co-regulation Network Rewiring
Source: BMC Bioinformatics. 2020 Jul 2;21:281. doi: 10.1186/s12859-020-03605-3 (PMC7333332; doi:10.1186/s12859-020-03605-3)
Supplement: Supplementary file 1 — Additional file 1. [file 12859_2020_3605_MOESM1_ESM.docx]

Supplementary file to

“DiNeR: a nonparametric *Di*fferential Graphical Model on *Ne*twork *R*ewiring to Infer Transcription Factor Co-binding Alterations in Disease”

# Rationale to match K562 to GM12878

Exact matching was not possible with K562: this cancer cell-line was derived from a myeloid lineage, but there is no data-rich, non-cancerous myeloid cell assayed in ENCODE. GM12878 is a data-rich ENCODE cell-line derived from the closely related lymphoid lineage. Supporting this choice, we determined that among all non-cancerous cell-lines provided by Roadmap Epigenome and GTEx, GM12878 has the highest Spearman correlation with K562 in gene expression. Hence, we used GM12878 as the most appropriate normal pair for K562.


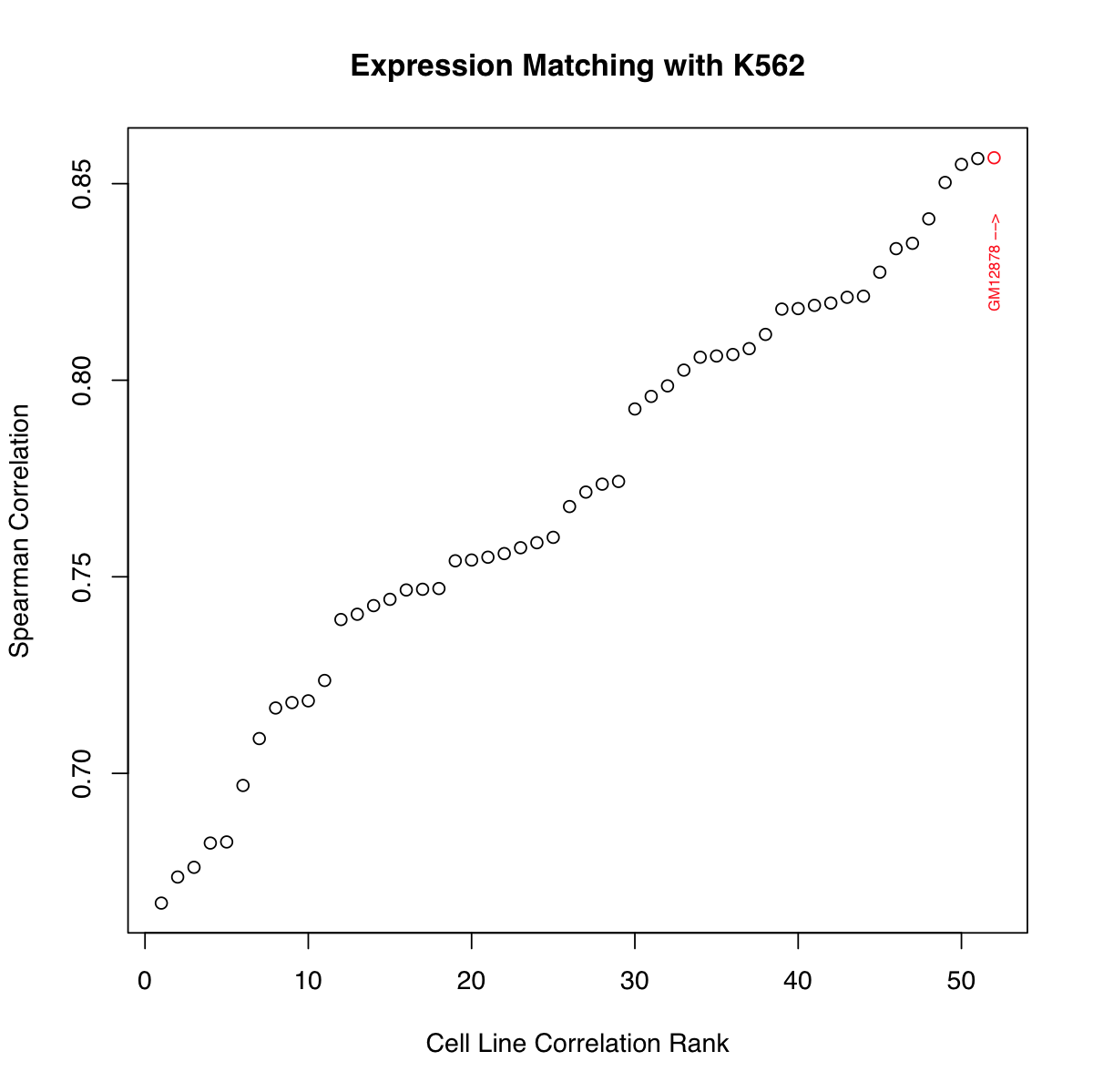


Figure S 1. Expression matching between cell types

# S3. Violation of normal assumption

We calculated the signal of each bin for all uniformly processed ChIP-seq data from ENCODE using bigWigAverageOverBed. Then, we took the log of the signal over the genome and plotted the density Figure S 2. We also plotted the QQ-plot vs. theoretical normal distribution, as shown in Figure S 3. The P-value of the Kolmogorov-Smirnov test for normality is less than $2.2e-16$, indicating a strong violation of the normal assumption.


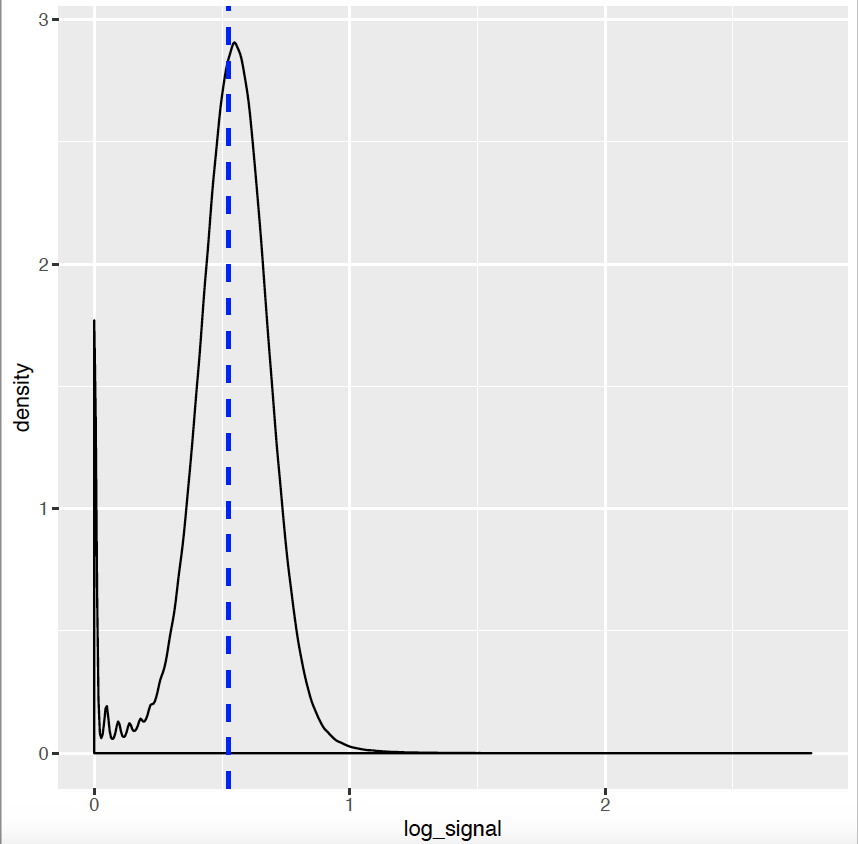


Figure S 2. density plot of the ChIP-seq signals


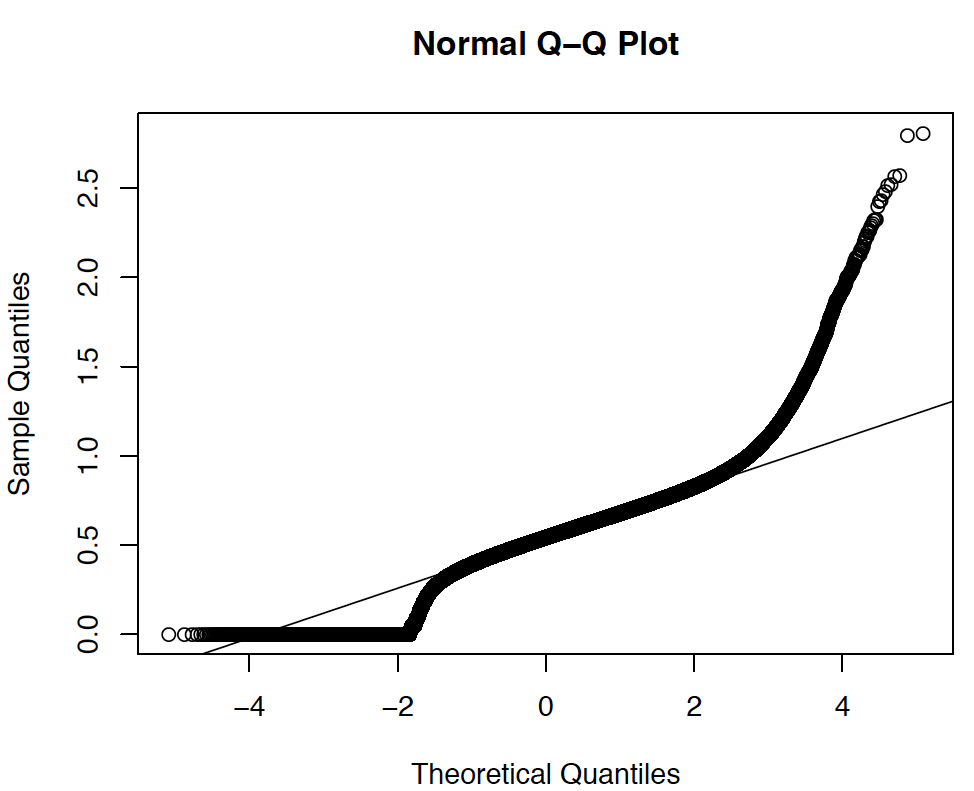


Figure S 3. Q-Q plot of the log transformed signals for ChIP-seq data

# BRCA1 survival analysis

Since BRCA1 demonstrates the highest number of rewired edges, as well as other significant regulatory changes between cancer and normal, we used the regulatory activity of BRCA1 as a marker for survival in acute myeloid leukemia (AML). We collected the cancer FPKM (Fragments Per Kilobase of transcript per Million mapped reads) expression data from 151 patients with AML from TCGA. In addition, we also collected the clinical information of each of these patients. Specifically, for deceased patients we used the number of days to death after diagnosis, while for alive patients, we used the days until the last follow up as a censored set of data for our survival analysis. We further compared the expression data for these cancer patients to a normal set of expression data from the GTex normal, whole blood cells. We scaled and took the difference between the cancer expression of each patient and the normal expression data in order to calculate the differential expression of each protein coding gene for each of the 151 patients. We extracted the network linking BRCA1 to over 2600 of the 20,000 protein coding genes, creating a binary vector of genes inside and outside of the BRCA1 network (represented by 1 and 0 respectively). A logistic regression was computed for each patient between this network vector and the set of differential expressions of each gene for each patient. The resulting coefficient for the regression was used as the regulatory activity for BRCA1 for a given patient. We then stratified the group of 151 patients by their high and low status of the BRCA1 regulatory activity. By stratifying the group in this way, we determine a statistically meaningful way of separating patients by quality of their prognosis.

# network centrality analysis

After selecting the optimal Lasso parameter $\lambda_{opt}$, we obtain the differential graphical model. In our case, we used $\lambda_{opt}=0.2$. We then extracted all the connected edges in the graph $G$ and calculated the number of edges per node in $G$. We selected the top 10 nodes with the most edges as the network hubs and calculated the percentage of the network that includes each node in the 100 sub-sampled networks under $\lambda_{opt}$. In the end, we selected 8 out of the 10 nodes as consistent network hubs with inclusion rate of over 90 percent.

Table S1. List of Factors shared by GM12878 and K562 used in analysis


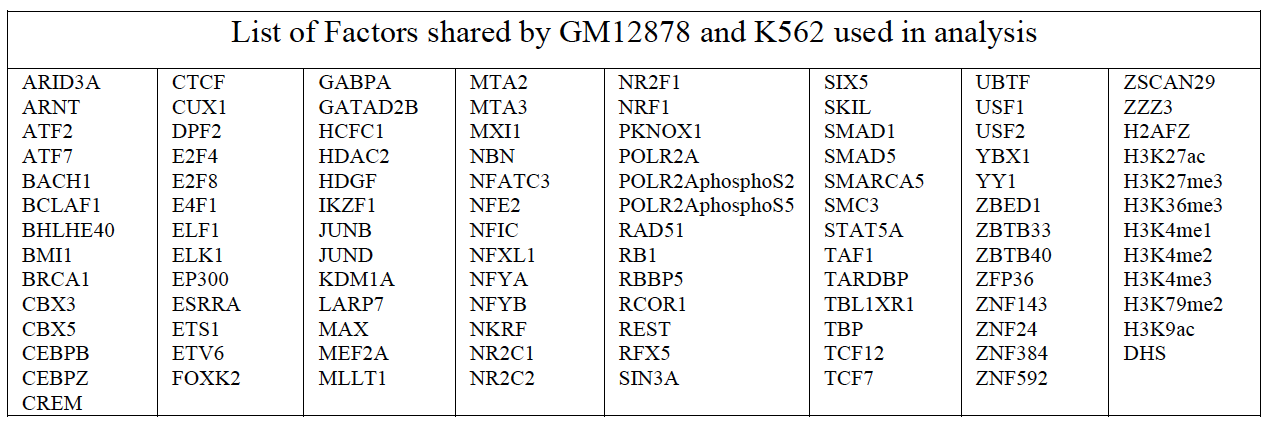


# Pseudocode for model selection

# genome sampling and differential network estimation

for $s$ in 1:$S$:

Sample half of the columns from $\boldsymbol{X}^{\left( 0 \right)}$ as $\boldsymbol{X}^{\left( 0 \right), s}$

Sample half of the columns from $\boldsymbol{X}^{\left( 1 \right)}$ as $\boldsymbol{X}^{\left( 1 \right), s}$

for $m$ in $1:M$:

given $\lambda_{m}$, estimate differential network $\boldsymbol{N}_{m}^{s}$ with edge between TFs $i$ and $j$ if $\left( \psi_{m}^{s} \right)_{ij}\neq0$

# genome sampling and differential network stability estimation

for $m$ in $1:M$:

$\hat{D}\left( \Lambda_{m} \right)= \hat{D}\left( 1/{\lambda_{m}} \right)=0$

for each possible edge $i,j (i<j)$ in $\left( N_{m}^{1}, \cdots, N_{m}^{s}, \cdots,N_{m}^{S} \right)$ $:$

$\theta_{i,j}\left( \Lambda_{m} \right)=\frac{1}{S}\sum_{s=1}^{S} \psi_{i,j}^{s}\left( \Lambda_{m} \right)$

$\xi_{i,j}\left( \Lambda_{m} \right)=2\theta_{i,j}\left( \Lambda_{m} \right)\left\{ 1-\theta_{i,j}\left( \Lambda_{m} \right) \right\}$

$\hat{D}\left( \Lambda_{m} \right)= \hat{D}\left( \Lambda_{m} \right)+\xi_{i,j}\left( \Lambda_{m} \right)$

$\hat{D}\left( \Lambda_{m} \right)=\frac{D\left( \Lambda_{m} \right)}{\left( \begin{matrix} J \\ 2 \end{matrix} \right)}$

# monotone network stability

for $m$ in $1:M$:

$\bar{D}\left( \Lambda_{m} \right)={sup}_{0\ll t\ll\lambda_{m}}\hat{D}\left( \Lambda_{m} \right)$

# optimal $\lambda$ selection

$\hat{\Lambda}_{opt}=sup\left\{ \Lambda:\bar{D}\left( \Lambda\right)\leq\beta\right\}$

# ChIP-Seq ENCODE Accession

## ENCODE K562 TF ChIP-Seq Accession codes

3xFLAG-ATF1 ENCSR159OCC

3xFLAG-PBX2 ENCSR633EIC

3xFLAG-ZNF146 ENCSR713IFY

3xFLAG-ZNF3 ENCSR195QFV

AFF1 ENCSR241LIH

AFF1 ENCSR426URK

AGO1 ENCSR641BSL

ARHGAP35 ENCSR571BUF

ARID1B ENCSR822CCM

ARID2 ENCSR491EBY

ARID3A ENCSR000EFY

ARNT ENCSR155KHM

ARNT ENCSR613NUC

ARNT ENCSR669NFS

ASH1L ENCSR115BBC

ATF1 ENCSR000DNZ

ATF2 ENCSR869IUD

ATF3 ENCSR000DOG

ATF3 ENCSR000BNU

ATF3 ENCSR028UIU

ATF4 ENCSR145TSJ

ATF7 ENCSR972ZBV

BACH1 ENCSR000EGD

BCLAF1 ENCSR000BKH

BCLAF1 ENCSR000BKH

BCLAF1 ENCSR492LTS

BCLAF1 ENCSR792IYC

BCOR ENCSR808AKZ

BDP1 ENCSR000DOK

BHLHE40 ENCSR000EGV

BMI1 ENCSR782WRO

BRCA1 ENCSR223MLH

BRD4 ENCSR583ACG

BRD9 ENCSR177XCS

BRF1 ENCSR000DOJ

BRF2 ENCSR000DOC

C11orf30 ENCSR350XWY

CBFA2T2 ENCSR699PVC

CBFA2T3 ENCSR697YLJ

CBX1 ENCSR948QLZ

CBX3 ENCSR000ATV

CBX3 ENCSR000BRT

CBX5 ENCSR272JAT

CBX8 ENCSR000ATW

CC2D1A ENCSR343IFJ

CCAR2 ENCSR598GER

CCNT2 ENCSR000DOA

CDC5L ENCSR121PFY

CEBPB ENCSR000EHE

CEBPZ ENCSR618GDK

CHAMP1 ENCSR065XVO

CHAMP1 ENCSR315NNL

COPS2 ENCSR130HEG

CREB3L1 ENCSR109YGM

CREBBP ENCSR000ATT

CREM ENCSR077DKV

CSDE1 ENCSR402XII

CSDE1 ENCSR626QJQ

CTBP1 ENCSR201NQZ

CTCFL ENCSR000BNK

CTCF ENCSR000AKO

CTCF ENCSR000DMA

CTCF ENCSR000DWE

CTCF ENCSR000BPJ

CTCF ENCSR000EGM

CTCF ENCSR000DWE

CTCF ENCSR000DWE

CUX1 ENCSR000EFO

DACH1 ENCSR030TJP

DDX20 ENCSR446LAV

DEAF1 ENCSR387SYS

DNMT1 ENCSR987PBI

DPF2 ENCSR219BXP

DPF2 ENCSR715CCR

E2F1 ENCSR563LLO

E2F1 ENCSR720HUL

E2F6 ENCSR000BLI

E2F7 ENCSR171CAY

E2F8 ENCSR953DVM

E4F1 ENCSR731LHZ

eGFP-ADNP ENCSR440VKE

eGFP-ATF1 ENCSR091GVJ

eGFP-ATF3 ENCSR632DCH

eGFP-BACH1 ENCSR740NPG

eGFP-CEBPB ENCSR416QLJ

eGFP-CEBPG ENCSR490LWA

eGFP-CEBPG ENCSR620VIC

eGFP-CREB3 ENCSR093FKD

eGFP-CUX1 ENCSR178NTX

eGFP-DDX20 ENCSR382AIB

eGFP-DIDO1 ENCSR167JBG

eGFP-E2F5 ENCSR709DRM

eGFP-ELF1 ENCSR975SSR

eGFP-ETS2 ENCSR596IKD

eGFP-ETV1 ENCSR277DMR

eGFP-FOSL1 ENCSR239ZLZ

eGFP-FOXJ2 ENCSR847LBF

eGFP-GABPA ENCSR290MUH

eGFP-GATA2 ENCSR000DKA

eGFP-GTF2A2 ENCSR801RPW

eGFP-GTF2E2 ENCSR532KTI

eGFP-HDAC8 ENCSR000DJZ

eGFP-HDAC8 ENCSR835TCD

eGFP-HINFP ENCSR619GFP

eGFP-ID3 ENCSR005NMT

eGFP-ILK ENCSR648INT

eGFP-IRF1 ENCSR854MCV

eGFP-IRF9 ENCSR926KTP

eGFP-KLF13 ENCSR608HVP

eGFP-KLF1 ENCSR550HCT

eGFP-MAFG ENCSR818DQV

eGFP-MEF2D ENCSR647ZXA

eGFP-NFE2L1 ENCSR632SHZ

eGFP-NFE2 ENCSR552YGL

eGFP-NR2C1 ENCSR178DEG

eGFP-NR2C2 ENCSR750LYM

eGFP-NR4A1 ENCSR000DJW

eGFP-NR4A1 ENCSR130PDE

eGFP-PBX2 ENCSR263DFP

eGFP-POLR2H ENCSR400FSM

eGFP-PTRF ENCSR126FZN

eGFP-PTTG1 ENCSR314BBS

eGFP-PYGO2 ENCSR410DWC

eGFP-RELA ENCSR772EEN

eGFP-TAF7 ENCSR671GFC

eGFP-TEAD2 ENCSR635GTR

eGFP-TFDP1 ENCSR017GBO

eGFP-TSC22D4 ENCSR787RVK

eGFP-USF2 ENCSR359NFW

eGFP-VEZF1 ENCSR189YMA

eGFP-ZBTB11 ENCSR706BJO

eGFP-ZBTB40 ENCSR158RYZ

eGFP-ZFX ENCSR920ASP

eGFP-ZKSCAN8 ENCSR448UKK

eGFP-ZNF148 ENCSR018MSO

eGFP-ZNF175 ENCSR011PEI

eGFP-ZNF197 ENCSR580IAO

eGFP-ZNF24 ENCSR117WTM

eGFP-ZNF354B ENCSR674SCQ

eGFP-ZNF395 ENCSR462QZZ

eGFP-ZNF507 ENCSR598TIR

eGFP-ZNF512 ENCSR591CCL

eGFP-ZNF584 ENCSR149ZBI

eGFP-ZNF589 ENCSR603XLW

eGFP-ZNF639 ENCSR845BCL

eGFP-ZNF644 ENCSR729HVR

eGFP-ZNF740 ENCSR532EMP

eGFP-ZNF740 ENCSR737UST

eGFP-ZNF766 ENCSR194IJN

eGFP-ZNF83 ENCSR257XVY

EGR1 ENCSR000BNE

EGR1 ENCSR024CNP

EGR1 ENCSR211LTF

EHMT2 ENCSR175EOM

ELF1 ENCSR000BMD

ELF1 ENCSR502OEK

ELF4 ENCSR638QHV

ELK1 ENCSR000EFU

EP300 ENCSR000EGE

EP400 ENCSR817QKV

ESRRA ENCSR486IFJ

ETS1 ENCSR000BKQ

ETV6 ENCSR000FCE

ETV6 ENCSR124BJR

EWSR1 ENCSR142YYA

FIP1L1 ENCSR177DNR

FOSL1 ENCSR000BMV

FOS ENCSR000FAI

FOXA1 ENCSR819LHG

FOXK2 ENCSR302AWT

FOXK2 ENCSR508DQA

FOXM1 ENCSR429QPP

FUS ENCSR051DXE

GABPA ENCSR000BLO

GABPB1 ENCSR138YYY

GATA1 ENCSR000EFT

GATA1 ENCSR000EWM

GATA2 ENCSR000EWG

GATAD2A ENCSR160QYK

GATAD2B ENCSR547LKC

GMEB1 ENCSR928KOR

GTF2B ENCSR000DOE

GTF2F1 ENCSR000EHC

GTF2F1 ENCSR189VXS

GTF2F1 ENCSR377BLZ

GTF3C2 ENCSR000DOD

HCFC1 ENCSR000EFN

HDAC1 ENCSR000AQF

HDAC1 ENCSR387UWP

HDAC1 ENCSR568PGX

HDAC1 ENCSR711VWL

HDAC2 ENCSR000AQG

HDAC2 ENCSR000BMG

HDAC2 ENCSR075HTM

HDAC2 ENCSR893WSB

HDAC3 ENCSR024LKA

HDAC6 ENCSR000ATJ

HDGF ENCSR197ALX

HDGF ENCSR563YDA

HES1 ENCSR091JXL

HLTF ENCSR090JNM

HMBOX1 ENCSR757IIU

HMGN3 ENCSR000DOB

HNRNPH1 ENCSR581CVA

HNRNPK ENCSR014RCS

HNRNPLL ENCSR112RNT

HNRNPL ENCSR594BNR

HNRNPUL1 ENCSR296MXW

IKZF1 ENCSR395HWC

IKZF1 ENCSR948VFL

ILF3 ENCSR632TJQ

IRF2 ENCSR376WCJ

JUNB ENCSR795IYP

JUND ENCSR000EGN

JUN ENCSR000FAH

JUN ENCSR000EFS

KAT2B ENCSR000ATZ

KAT8 ENCSR086FZL

KDM1A ENCSR360HRA

KDM1A ENCSR908CMW

KDM4B ENCSR642VZY

KDM4B ENCSR660RNO

KDM5B ENCSR000AQA

KHSRP ENCSR686EYO

KLF16 ENCSR760UVO

L3MBTL2 ENCSR530XQI

LARP7 ENCSR288MOZ

LEF1 ENCSR343ELW

LEF1 ENCSR832OGB

MAFF ENCSR000EGI

MAFK ENCSR000EGX

MAX ENCSR000BLP

MAX ENCSR000EFV

MBD2 ENCSR221GAN

MCM2 ENCSR138SFL

MCM2 ENCSR552PSV

MCM3 ENCSR990AZC

MCM5 ENCSR079WHK

MCM5 ENCSR628APV

MCM7 ENCSR038RGL

MCM7 ENCSR068VRA

MCM7 ENCSR542WJU

MEF2A ENCSR000BNV

MEIS2 ENCSR851BNE

MGA ENCSR710WLO

MIER1 ENCSR426MDV

MITF ENCSR000FCB

MITF ENCSR797SWM

MLLT1 ENCSR107GRP

MLLT1 ENCSR675LRO

MNT ENCSR390VGH

MNT ENCSR512NLO

MNT ENCSR979QYJ

MTA1 ENCSR807BGP

MTA2 ENCSR113LAS

MTA2 ENCSR411UYA

MTA3 ENCSR914NEI

MXI1 ENCSR000EGZ

MYBL2 ENCSR162IEM

MYC ENCSR000FAG

MYC ENCSR000EGJ

MYNN ENCSR737LTZ

NBN ENCSR085QEV

NCOA1 ENCSR658WFQ

NCOA1 ENCSR711SNW

NCOA1 ENCSR931HNY

NCOA2 ENCSR349TZO

NCOA2 ENCSR803EKW

NCOA4 ENCSR119ULQ

NCOA6 ENCSR168CEE

NCOR1 ENCSR298JCG

NCOR1 ENCSR798ILC

NCOR1 ENCSR910JAI

NELFE ENCSR000DOF

NEUROD1 ENCSR986CDX

NFATC3 ENCSR051OUX

NFATC3 ENCSR670FDA

NFE2 ENCSR000FAF

NFE2 ENCSR000FCC

NFIC ENCSR796ITY

NFRKB ENCSR657EOF

NFRKB ENCSR996ESX

NFXL1 ENCSR085DDI

NFYA ENCSR000EGR

NFYB ENCSR000EGQ

NKRF ENCSR331BDJ

NONO ENCSR415TXN

NONO ENCSR886RYH

NR0B1 ENCSR764YDL

NR2C1 ENCSR742IDN

NR2C2 ENCSR000EWH

NR2F1 ENCSR970NKQ

NR2F2 ENCSR000BRS

NR2F6 ENCSR707QWA

NR3C1 ENCSR325DER

NR3C1 ENCSR494UQJ

NRF1 ENCSR494TDU

NRF1 ENCSR837EYC

NRF1 ENCSR998AJK

NUFIP1 ENCSR574XEO

PCBP1 ENCSR052PTN

PCBP2 ENCSR603REQ

PHB2 ENCSR924GXX

PHF20 ENCSR594SMP

PHF21A ENCSR119VCX

PHF8 ENCSR000AQH

PKNOX1 ENCSR115SMW

PML ENCSR000BQY

POLR2A ENCSR000FAJ

POLR2A ENCSR000BMR

POLR2A ENCSR000EHL

POLR2A ENCSR031TFS

POLR2A ENCSR388QZF

POLR2AphosphoS2 ENCSR000EHF

POLR2AphosphoS2 ENCSR000EGF

POLR2AphosphoS5 ENCSR000BKR

POLR2B ENCSR325RLL

POLR2G ENCSR283ZRI

POLR3A ENCSR000DOI

POLR3G ENCSR000EHQ

POU5F1 ENCSR364SNE

PRDM10 ENCSR120MPG

PRPF4 ENCSR220YXI

PTBP1 ENCSR948KMB

PYGO2 ENCSR431XGJ

RAD21 ENCSR000FAD

RAD51 ENCSR524BUE

RB1 ENCSR670JDQ

RBBP5 ENCSR000AQI

RBFOX2 ENCSR822LBD

RBM14 ENCSR423FCW

RBM15 ENCSR850WUE

RBM17 ENCSR836COE

RBM22 ENCSR848AOP

RBM25 ENCSR791OZM

RBM34 ENCSR899GSH

RBM39 ENCSR764OXF

RCOR1 ENCSR000EGC

REST ENCSR000BMW

REST ENCSR137ZMQ

RFX1 ENCSR041AXL

RFX1 ENCSR968GIB

RFX5 ENCSR000EGO

RLF ENCSR718SDE

RNF2 ENCSR076YPO

RNF2 ENCSR138FUZ

RNF2 ENCSR608XTF

RNF2 ENCSR820GND

RUNX1 ENCSR414TYY

RUNX1 ENCSR588AKU

SAFB2 ENCSR724GSW

SAFB ENCSR072VUO

SAP30 ENCSR000AQJ

SETDB1 ENCSR000AUT

SETDB1 ENCSR000EWI

SFPQ ENCSR647PJW

SIN3A ENCSR000BLR

SIN3A ENCSR920BLG

SIN3B ENCSR657JLK

SIRT6 ENCSR000DOH

SIRT6 ENCSR000AUB

SIX5 ENCSR000BGX

SIX5 ENCSR000BNW

SKIL ENCSR336DXE

SMAD1 ENCSR038DJJ

SMAD2 ENCSR189PYJ

SMAD5 ENCSR000FCD

SMARCA4 ENCSR000EHO

SMARCA4 ENCSR587OQL

SMARCA4 ENCSR643VTW

SMARCA5 ENCSR895HSJ

SMARCB1 ENCSR000EHN

SMARCC2 ENCSR519WMW

SMARCE1 ENCSR157TCS

SMC3 ENCSR000EGW

SNIP1 ENCSR654CQU

SNRNP70 ENCSR754ZHU

SOX6 ENCSR788RSW

SP1 ENCSR991ELG

SPI1 ENCSR000BGW

SREBF1 ENCSR815ZDS

SRSF1 ENCSR832DMO

SRSF3 ENCSR268QIQ

SRSF7 ENCSR222MYK

SRSF9 ENCSR814AJH

STAT5A ENCSR000BRR

SUPT5H ENCSR894CGX

SUZ12 ENCSR000AUC

SUZ12 ENCSR412CTM

TAF15 ENCSR047LSJ

TAF1 ENCSR000BKS

TAF7 ENCSR000BNM

TAF9B ENCSR100UQX

TAL1 ENCSR000EHB

TAL1 ENCSR106FRG

TARDBP ENCSR033VAZ

TARDBP ENCSR353HEP

TARDBP ENCSR429XTR

TBL1XR1 ENCSR000EGA

TBL1XR1 ENCSR000EGB

TBP ENCSR000EHA

TCF12 ENCSR189TRZ

TCF12 ENCSR744WOO

TCF7L2 ENCSR888XZK

TCF7 ENCSR863KUB

TEAD4 ENCSR000BRK

THAP1 ENCSR000BNN

THRA ENCSR264CZJ

THRAP3 ENCSR871TKJ

TOE1 ENCSR213HBY

TRIM24 ENCSR907MZR

TRIM24 ENCSR957LDM

TRIM25 ENCSR213VUI

TRIM25 ENCSR664AOA

TRIM28 ENCSR000BRW

TRIM28 ENCSR000EVY

TRIM28 ENCSR474CVP

TRIP13 ENCSR154EIH

U2AF1 ENCSR690GUG

U2AF2 ENCSR479QAJ

UBTF ENCSR000EFW

UBTF ENCSR000EFZ

USF1 ENCSR000BKT

USF2 ENCSR000EHG

WHSC1 ENCSR000AVE

XRCC3 ENCSR997NGQ

XRCC5 ENCSR506KWJ

YBX1 ENCSR107RHZ

YBX3 ENCSR567JEU

YY1 ENCSR000BKU

YY1 ENCSR000BMH

YY1 ENCSR000EWF

ZBED1 ENCSR286PCG

ZBTB11 ENCSR331GDC

ZBTB2 ENCSR230PTV

ZBTB33 ENCSR876GXA

ZBTB40 ENCSR237VLT

ZBTB5 ENCSR389PWB

ZBTB5 ENCSR786OQY

ZBTB7A ENCSR000BME

ZBTB8A ENCSR283ZNI

ZC3H11A ENCSR000EFR

ZC3H8 ENCSR143CEO

ZC3H8 ENCSR494PWZ

ZEB2 ENCSR004GKA

ZEB2 ENCSR322CFO

ZFP36 ENCSR776CYN

ZFP91 ENCSR898XMH

ZHX1 ENCSR557RVF

ZKSCAN1 ENCSR882ERE

ZMIZ1 ENCSR907JPB

ZMYM3 ENCSR102KIN

ZNF143 ENCSR000EGP

ZNF184 ENCSR546IHU

ZNF184 ENCSR621ATC

ZNF24 ENCSR099NCH

ZNF24 ENCSR385AHH

ZNF24 ENCSR695EQB

ZNF263 ENCSR000EWN

ZNF274 ENCSR000EVX

ZNF274 ENCSR000EWE

ZNF280A ENCSR370NFS

ZNF282 ENCSR742TMU

ZNF316 ENCSR167KBO

ZNF316 ENCSR200JYP

ZNF318 ENCSR334HSW

ZNF318 ENCSR352BJL

ZNF384 ENCSR000EFP

ZNF407 ENCSR011NOZ

ZNF407 ENCSR439OCL

ZNF592 ENCSR249BHQ

ZNF639 ENCSR497VFH

ZNF639 ENCSR949NVY

ZNF830 ENCSR033NQK

ZNF830 ENCSR851XLW

ZSCAN29 ENCSR175SZH

ZSCAN29 ENCSR635EXI

ZZZ3 ENCSR780BBJ

## ENCODE GM12878 TF ChIP-Seq Accession codes

ARID3A ENCSR778UBR

ARNT ENCSR590KEQ

ASH2L ENCSR849WCQ

ATF2 ENCSR000BQK

ATF2 ENCSR961PPA

ATF7 ENCSR014YCR

BACH1 ENCSR636MKU

BATF ENCSR000BGT

BCL11A ENCSR000BHA

BCL3 ENCSR000BNQ

BCLAF1 ENCSR000BJZ

BCLAF1 ENCSR342THD

BHLHE40 ENCSR000DZJ

BHLHE40 ENCSR987MTA

BMI1 ENCSR469WII

BRCA1 ENCSR000DZS

CBFB ENCSR860UHK

CBX3 ENCSR549NPZ

CBX5 ENCSR372GIN

CEBPB ENCSR681NOM

CEBPZ ENCSR347NOB

CHD1 ENCSR000DZE

CHD2 ENCSR000DZR

CHD4 ENCSR751CJG

CREM ENCSR839XZU

CTCF ENCSR000DKV

CTCF ENCSR000AKB

CTCF ENCSR000DRZ

CTCF ENCSR000DZN

CUX1 ENCSR000DYR

DPF2 ENCSR509FWH

E2F4 ENCSR000DYY

E2F8 ENCSR793HVL

E4F1 ENCSR439WAF

EBF1 ENCSR000BGU

EBF1 ENCSR000DZQ

EED ENCSR199WXF

EGR1 ENCSR000BRG

ELF1 ENCSR841NDX

ELK1 ENCSR000DZB

EP300 ENCSR000BHB

EP300 ENCSR000DZD

EP300 ENCSR000DZG

ESRRA ENCSR000DYQ

ETS1 ENCSR000BKA

ETV6 ENCSR597VGC

ETV6 ENCSR626VUC

EZH2 ENCSR000ARD

FOS ENCSR000EYZ

FOXK2 ENCSR861JUQ

GABPA ENCSR331HPA

GATAD2B ENCSR828NCB

HCFC1 ENCSR514VAY

HDAC2 ENCSR330OEO

HDAC6 ENCSR933EYC

HDGF ENCSR145XQO

HSF1 ENCSR009MBP

IKZF1 ENCSR000EUJ

IKZF1 ENCSR441VHN

IKZF1 ENCSR874AFU

IKZF2 ENCSR680UQE

IKZF2 ENCSR822AHX

IRF3 ENCSR000DZX

IRF3 ENCSR408JQO

IRF4 ENCSR000BGY

IRF5 ENCSR976TBC

JUNB ENCSR897MMC

JUND ENCSR000EYV

JUND ENCSR000DYS

KDM1A ENCSR391IWM

KLF5 ENCSR974OFJ

LARP7 ENCSR657PEW

MAFK ENCSR000DYV

MAX ENCSR000DZF

MAZ ENCSR000DZA

MEF2A ENCSR000BKB

MEF2B ENCSR177VFS

MEF2C ENCSR000BNG

MLLT1 ENCSR552XSN

MTA2 ENCSR293QAR

MTA3 ENCSR000BRH

MXI1 ENCSR000DZI

MYB ENCSR819ATC

MYC ENCSR000DKU

NBN ENCSR278SQL

NFATC1 ENCSR000BQL

NFATC3 ENCSR437GBJ

NFE2 ENCSR000DZY

NFIC ENCSR000BRN

NFXL1 ENCSR746XEG

NFYA ENCSR000DNN

NFYB ENCSR000DNM

NKRF ENCSR732PJX

NR2C1 ENCSR784VIQ

NR2C2 ENCSR000EUL

NR2F1 ENCSR514VYD

NRF1 ENCSR000DZO

PAX5 ENCSR000BHD

PAX5 ENCSR000BHJ

PAX8 ENCSR192AFN

PBX3 ENCSR000BGR

PKNOX1 ENCSR711XNY

POLR2A ENCSR000BGD

POLR2A ENCSR000BGD

POLR2AphosphoS2 ENCSR000DZK

POLR2AphosphoS5 ENCSR000BIF

POLR3G ENCSR000EYU

RAD21 ENCSR000EAC

RAD21 ENCSR000BMY

RAD51 ENCSR482TWQ

RB1 ENCSR785OKZ

RBBP5 ENCSR330EXS

RCOR1 ENCSR000DZC

RELB ENCSR387QUV

REST ENCSR000BGF

REST ENCSR000BQS

RFX5 ENCSR000DZW

RUNX3 ENCSR000BRI

RXRA ENCSR000BJD

SIN3A ENCSR000DYX

SIX5 ENCSR000BJE

SKIL ENCSR212YKD

SMAD1 ENCSR813DCK

SMAD5 ENCSR251OVJ

SMARCA5 ENCSR706YUH

SMC3 ENCSR000DZP

SP1 ENCSR000BHK

SPI1 ENCSR000BGQ

SRF ENCSR000BGE

SRF ENCSR000BMI

SRF ENCSR041XML

STAT1 ENCSR332EYT

STAT3 ENCSR000DZV

STAT5A ENCSR000BQZ

SUZ12 ENCSR091BOQ

TAF1 ENCSR000BGS

TARDBP ENCSR016UEH

TARDBP ENCSR412QBS

TBL1XR1 ENCSR000DYZ

TBP ENCSR000DZZ

TBX21 ENCSR739IHN

TCF12 ENCSR000BGZ

TCF12 ENCSR725VFL

TCF7 ENCSR501DKS

TRIM22 ENCSR637QAM

TRIM22 ENCSR835XKS

UBTF ENCSR459FTB

USF1 ENCSR000BGI

USF2 ENCSR000DZU

WRNIP1 ENCSR000EAA

YBX1 ENCSR205SKQ

YY1 ENCSR000BNP

YY1 ENCSR000EUM

ZBED1 ENCSR207PFI

ZBTB33 ENCSR000BHC

ZBTB33 ENCSR542FLV

ZBTB40 ENCSR189YYK

ZEB1 ENCSR000BND

ZFP36 ENCSR900XDB

ZNF143 ENCSR000DZL

ZNF143 ENCSR936XTK

ZNF207 ENCSR117KWH

ZNF217 ENCSR764CZW

ZNF24 ENCSR072PWP

ZNF384 ENCSR000DYP

ZNF592 ENCSR173ZVL

ZNF622 ENCSR075FNZ

ZNF687 ENCSR859FDL

ZSCAN29 ENCSR412YGM

ZZZ3 ENCSR000DNQ
